# Urosepsis secondary to ureterosciatic hernia corrected with ureteral stent placement: a case report and literature review

**DOI:** 10.1186/s12245-021-00392-3

**Published:** 2021-11-06

**Authors:** Kohei Kakimoto, Mayu Hikone, Ko Nagai, Jun Yamakawa, Kazuhiro Sugiyama, Yuichi Hamabe

**Affiliations:** Tertiary Emergency Medical Center (Trauma and Critical Care), Tokyo Metropolitan Bokutoh Hospital, 4-23-15, Kotobashi, Sumida-ku, Tokyo, 130-8575 Japan

**Keywords:** Ureterosciatic hernia, Sepsis, Urinary tract infection, Ureteral catheter replacement

## Abstract

**Background:**

Ureterosciatic hernia is a rare type of pelvic floor herniation that occurs through the sciatic foramen. The resulting ureteral obstruction may lead to hydronephrosis and to further complications including urinary tract infection and urosepsis. There have been 30 reported cases of ureterosciatic hernia. Ureteral stenting and surgical repair have been used as treatment options.

**Case presentation:**

We report the case of an 86-year-old woman who was transferred to Tokyo Metropolitan Bokutoh Hospital with symptoms of fever and septic shock. Her computed tomography scan revealed left hydronephrosis and deviation of the left ureter into the sciatic foramen; she was therefore diagnosed with a left ureteral sciatic hernia and admitted in our intensive care unit for further treatment with resuscitative fluids, vasopressors, and antibiotics. Following a retrograde insertion ureteral catheter insertion, ureteral incarceration was relieved, and a double-J ureteral stent was placed in situ. Antibiotic treatment was initiated, and the patient’s hemodynamic status gradually improved.

**Conclusions:**

Although ureterosciatic hernia is a rare disorder, it is associated with serious complications including urinary tract infection with sepsis, which may warrant urgent corrective procedure to relieve the structural obstruction. Treatment may be conservative or surgical, though treatment with ureteral stent placement may be a favorable approach in elderly patients with multiple comorbidities presenting with urosepsis.

## Background

Ureterosciatic hernia is a relatively rare disorder that commonly occurs in elderly women, wherein the ureter herniates through the sciatic foramen [1]. This condition may lead to ureteral occlusion, and subsequent complications including hydronephrosis and urinary tract infection, which warrant urgent treatment to relieve the structural ureteral obstruction [2]. Although reports of conservative treatment by ureteral stent placement have increased in recent years, there are no fixed guidelines on determining a particular treatment approach such as surgery or stent placement. We report the case of a patient who developed urosepsis secondary to ureterosciatic hernia and who improved following ureteral stent placement. We also review the existing literature on the treatment of ureterosciatic hernia that will help determine a treatment strategy in affected, comorbid patients who may be hemodynamically unstable at presentation.

## Case presentation

An 86-year-old woman was transferred to the emergency and critical care center of Tokyo Metropolitan Bokutoh Hospital from a nearby general hospital with vital signs indicative of shock. She had been diagnosed with urinary tract infection. She had a medical history of chronic heart failure with pulmonary hypertension and was on home oxygen therapy for chronic respiratory failure.

On initial physical examination at arrival to our facility, she was conscious and oriented with a Glasgow Coma Scale score of 15 and a body temperature of 36.0 °C. On receiving a 0.3γ dose of noradrenaline, her blood pressure, pulse rate, and respiratory rate were maintained at 91/63 mmHg, 90 beats/min, and 24 breaths/min, respectively. Her oxygen saturation was 100% while receiving 10 L/min oxygen through a face mask. On physical examination, her left abdominal and left lumbar areas were tender. Her initial arterial blood gas analysis while on 10 L/min oxygen revealed a pH of 7.441, PaCO_2_ of 44.6 Â mmHg, PaO_2_ of 275.5 Â mmHg, HCO_3_^−^ of 29.9 mmol/L, SaO_2_ of 93.1%, and lactate of 1.3 mmol/L.

**Fig. 1:**
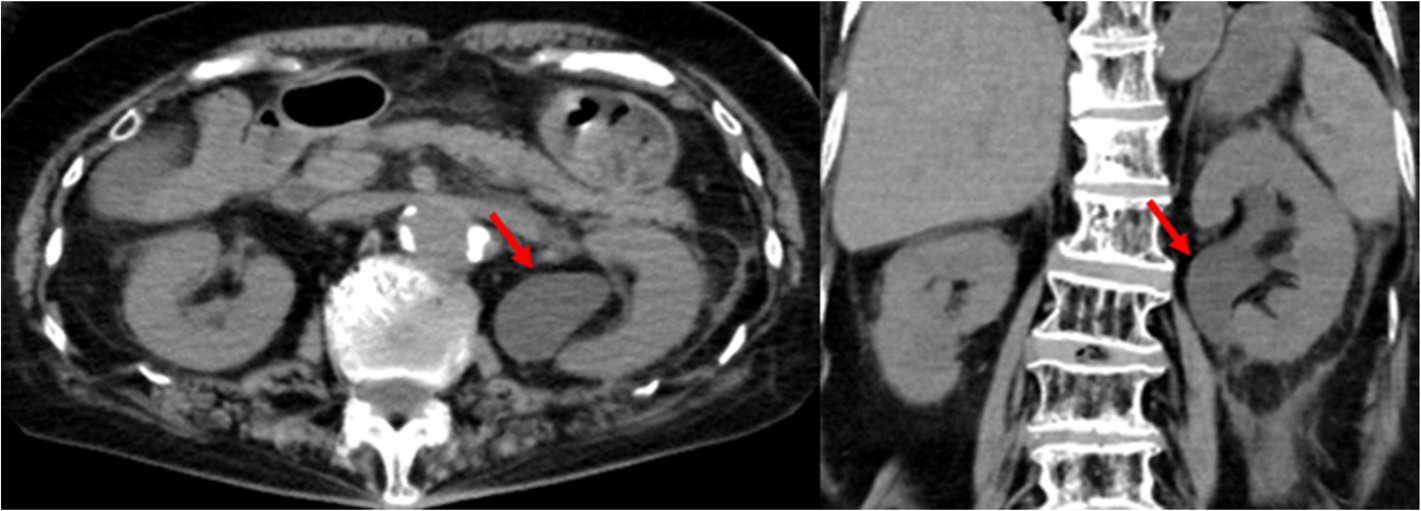
Computed tomography reveals left hydronephrosis with adipose tissue opacity around the left kidney

**Fig. 2:**
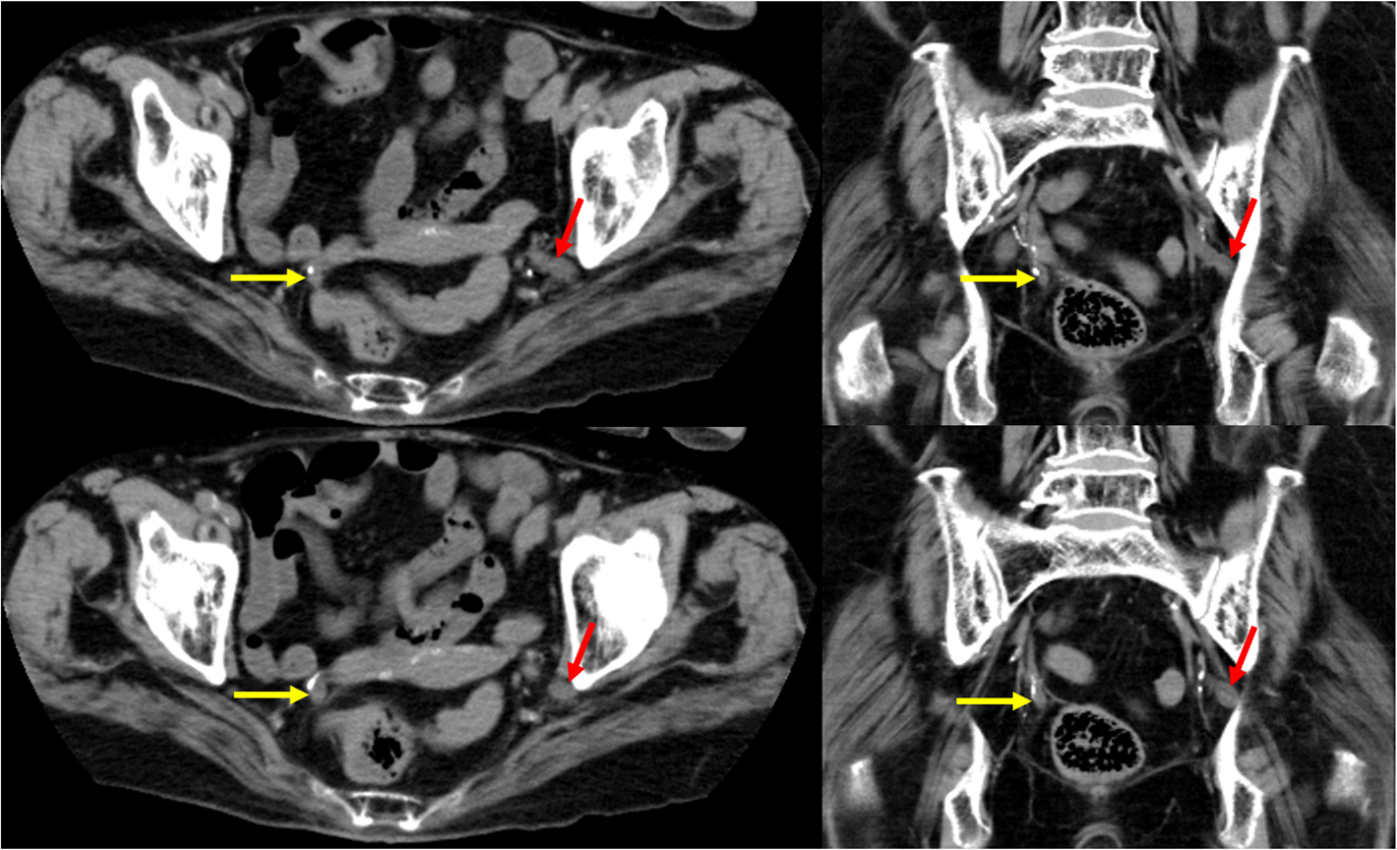
Computed tomography reveals deviation of the left ureter (red arrows) to the sciatic foramen. The right ureter is indicated by yellow arrows

**Fig. 3:**
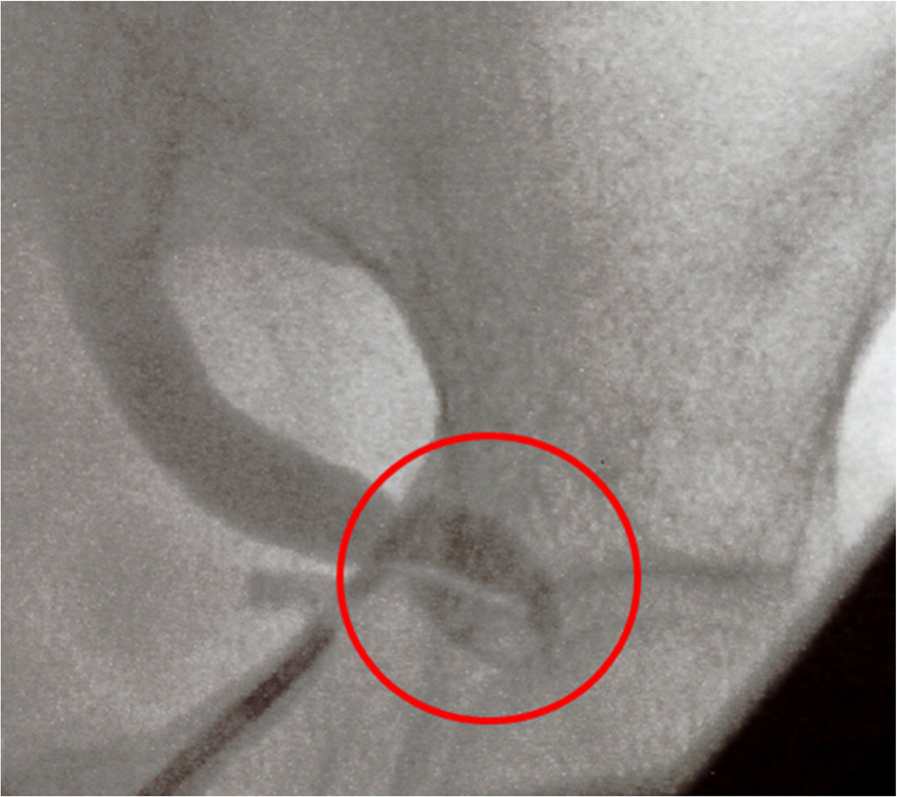
Retrograde urography reveals deviation of the left ureter into the sciatic foramen

**Fig. 4:**
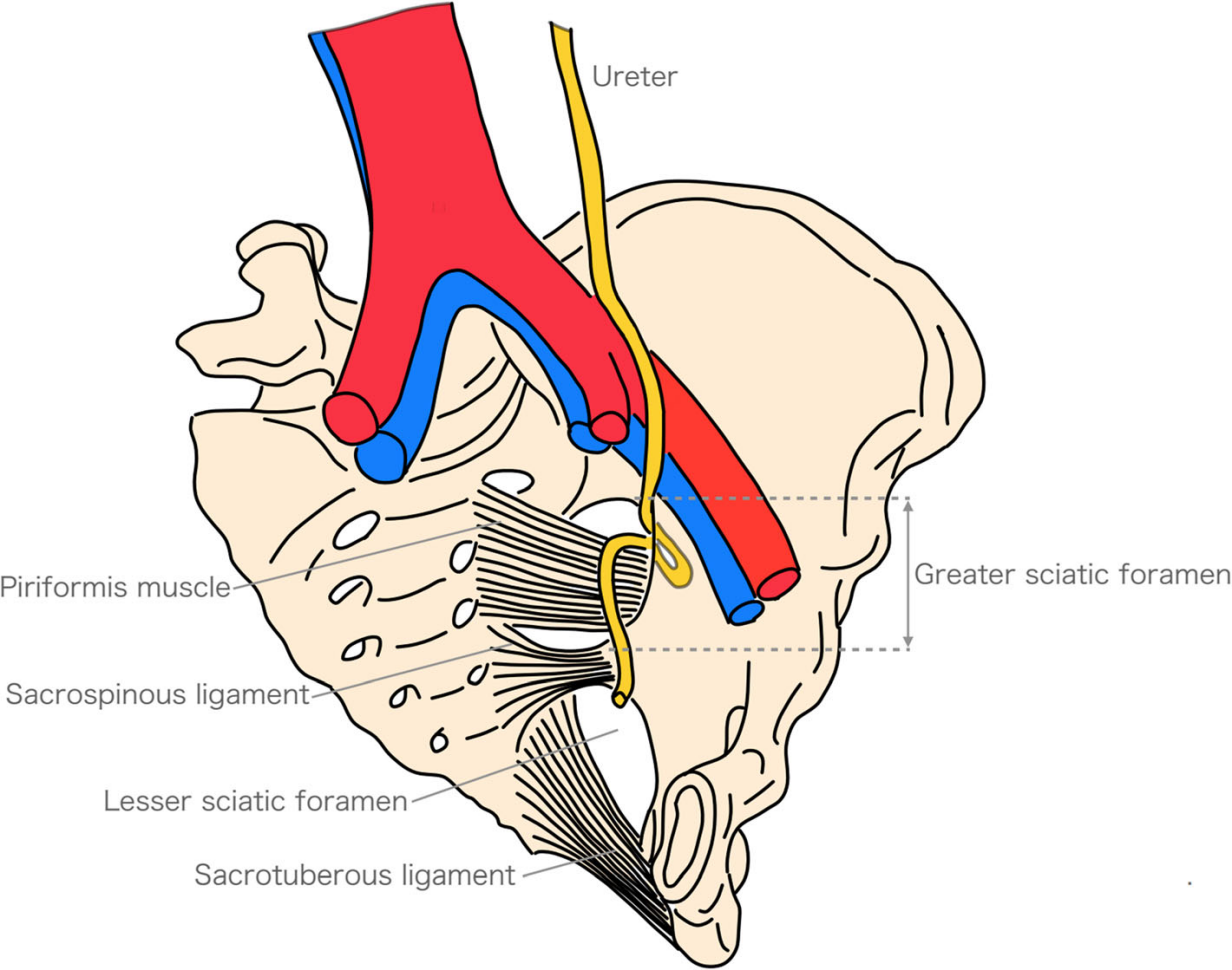
Ureterosciatic herniation, oblique view

The patient’s urine was negative for nitrite, white blood cells, and bacteria. Serum white blood cell count was 11,900/mm^3^, platelet count was 17.8 mm^3^, total bilirubin was 0.46 mg/dl, creatinine was 1.94 mg/dl, and C-reactive protein was 6.92 mg/dL. An unenhanced abdominal computed tomography scan revealed left hydronephrosis, adipose tissue opacity around the left kidney, and deviation of the left ureter to the sciatic foramen (Figs. 1 and 2). She was diagnosed with obstructive pyelonephritis associated with septic shock due to a ureterosciatic hernia. She was admitted to our intensive care unit for further treatment with resuscitative fluids, vasopressors, and antibiotics. As the patient was hemodynamically unstable, placement of a ureteral stent was attempted for relieving the herniation-associated structural obstruction. On a retrograde ureteral catheter insertion, the ureteral incarceration reduced, and we were able to place a double-J ureteral stent in situ (Figs. 3 and 4).

Initially, she required 0.35γ of noradrenaline and resuscitative extracellular fluids to maintain hemodynamic status. However, the day after the stent placement, she gradually recovered from septic shock and was tapered off vasopressors by day 4. Both blood and urine cultures revealed *Escherichia coli* with good antimicrobial sensitivity.

She continued to receive antibiotic treatment and underwent rehabilitation, with the goal of discharge and continued outpatient care. Unfortunately, she experienced exacerbation of respiratory failure and died on the 32nd day of hospitalization.

## Discussion and conclusions

While ureteral herniation is relatively uncommon, ureter prolapse into the inguinal canal and in the femoral canal may be observed, though the occurrence of sciatic herniation is extremely rare [3]. Elderly women are more susceptible, possibly because they experience increased abdominal pressure due to conditions such as a history of childbirth, wide pelvic opening, pregnancy, constipation, and age-related piriformis muscle atrophy and weakening [4]. In Japan, where nearly 30% of the population is 65 or older, it is necessary to distinguish this condition in elderly patients presenting with hydronephrosis and pyelonephritis. While computed tomography is useful for diagnosing ureterosciatic herniation, considering that it is a rare condition, the cause of obstruction may not be identifiable without including it in the differential diagnosis.

On searching the PubMed database using the keywords “ureterosciatic hernia” or “uretero sciatic hernia,” we identified 30 reported cases of ureterosciatic hernia since 1999, from English-language papers (Table 1) [1–3, 5–31]. The median patient age was 75.5 (57–97) years; all were female. The left side was affected in 22 patients (one bilateral disease). We speculated that the laterality was observed as the left ureter tends to be anatomically longer than the right ureter [32]. The initial therapeutic approaches used were stent placement in 21 patients, surgical repair in six patients, manual reposition in one patient, and simple observation without any treatment procedures in two patients. The initial attempt at ureteral stent placement was successful in 18/21 patients. Of these, 11 patients did not need additional procedures, while seven required further surgical treatment (in one case, surgery was planned in advance). Two patients treated solely with ureteral stenting relapsed after stent removal and also required surgical management. Of the total 16 patients who finally underwent surgical treatment, 12 patients underwent laparoscopic (four robot-assisted procedures), and four patients underwent open surgery.

**Table 1:** Clinical review of the reported cases of ureterosciatic hernia

Case no.	Author	Age	Sex	L/R	UTI	Shock	Initial treatment	Result of stent placement	Definitive treatment
1	Gee et al. [5]	60	F	L	–	–	Stenting	Recurrence after stent removal	Laparoscopic surgery
2	Weintraub et al. [6]	87	F	R	+	–	Stenting	Repaired	Stenting
3	Noller and Noller [7]	62	F	L	–	–	Stenting (failure)	–	Open surgery
4	Touloupidis et al. [8]	61	F	R	–	–	Surgery	–	Open surgery
5	Loffroy et al. [1]	81	F	L	–	–	Surgery	–	Open surgery
6	Tsai et al. [9]	91	F	L	–	–	Observation	–	Observation
7	Hsu et al. [10]	69	F	L	–	–	Stenting	Repaired	Stenting
8	Clemens et al. [11]	80	F	L	–	–	Stenting	Not repaired	Stenting (not repaired)
9	Sugimoto et al. [12]	76	F	L	–	–	Stenting	Repaired	Stenting
10	Whyburn and Alizadeh [13]	74	F	both	–	–	Stenting	Recurrence after stent removal	Laparoscopic surgery
11	Hemal et al. [14]	75	F	L	–	–	Stenting	Removed due to discomfort	Laparoscopic surgery
12	Tsuzaka et al. [15]	78	F	L	–	–	Surgery	–	Laparoscopic surgery
13	Kato et al. [16]	72	F	L	–	–	Stenting	Repaired	Stenting
14	Salari et al. [17]	87	F	R	–	–	Stenting	Repaired	Stenting
15	Yanagi et al. [3]	92	F	L	–	–	Stenting	Repaired	Stenting
16	Regelman and Raman [18]	60	F	L	–	–	Surgery	–	Laparoscopic surgery
17	Demetriou et al. [19]	76	F	L	–	–	Observation	–	Observation
18	Wai et al. [20]	68	F	L	–	–	Surgery	–	Laparoscopic surgery
19	Lin et al. [21]	81	F	R	–	–	Stenting	Not repaired	Open surgery
20	Nakazawa et al. [22]	92	F	L	–	–	Stenting	Repaired	Stenting
21	Fadel et al. [2]	65	F	R	+	+	Stenting	Repaired	Stenting
22	Destan and Durand [23]	80	F	R	+	–	Stenting + Surgery	Performed before surgery	Laparoscopic surgery
23	Moon et al. [24]	72	F	R	–	–	Stenting (failure)	–	Laparoscopic surgery
24	Kimura et al. [25]	86	F	L	–	–	Manual reposition	–	Manual reposition
25	Nagasubramanian et al. [26]	57	F	L	–	–	Stenting	Not repaired	Laparoscopic surgery
26	Kubota et al. [27]	85	F	L	+	+	Stenting	Removed due to discomfort	Laparoscopic surgery
27	Kim et al. [28]	68	F	L	–	–	Stenting	Repaired	Stenting
28	Kamisawa et al. [29]	70	F	R	–	–	Surgery	–	Laparoscopic surgery
29	Rose et al. [30]	68	F	L	–	–	Stenting (failure)	–	Laparoscopic surgery
30	Chan et al. [31]	97	F	L	+	–	Stenting	Repaired	Stenting

*F*, female; *L*, left; *R*, right; *UTI*, urinary tract infection

In our case, ureterosciatic herniation of the left ureter occurred in an elderly woman, which was consistent with previously reported clinical characteristics. Since the patient was elderly, had baseline respiratory dysfunction, and was hemodynamically unstable at the time of admission, she was considered a high-risk surgical candidate and was therefore treated using a stent. The ureteral obstruction improved after stenting. As the patient had chronic respiratory failure at baseline, the chronic increase in abdominal pressure due to respiratory failure may have contributed to the development of the ureterosciatic hernia.

In conclusion, we report the case of a patient presenting with ureterosciatic herniation and urosepsis, who improved after ureteral stent placement, and review the existing literature on this rare type of hernia. It is necessary to distinguish this underlying condition in elderly women with hydronephrosis and pyelonephritis. Although there is no established treatment strategy, we believe that stent placement is a better approach in comorbid, elderly patients who may be hemodynamically unstable at presentation and may therefore be unable to tolerate corrective surgery.
